# Bioinspired Membrane Interfaces: Controlling Actomyosin Architecture and Contractility

**DOI:** 10.1021/acsami.3c00061

**Published:** 2023-02-27

**Authors:** Nils L. Liebe, Ingo Mey, Loan Vuong, Fadi Shikho, Burkhard Geil, Andreas Janshoff, Claudia Steinem

**Affiliations:** † Institut für Organische und Biomolekulare Chemie, Georg-August Universität, Tammannstr. 2, Göttingen 37077, Germany; ‡ Institut für Physikalische Chemie, Georg-August Universität, Tammannstr. 6, Göttingen 37077, Germany; § Max-Planck-Institut für Dynamik und Selbstorganisation, Am Fassberg 17, Göttingen 37077, Germany

**Keywords:** actin, ERM proteins, fluorescence microscopy, myosin, supported lipid bilayers

## Abstract

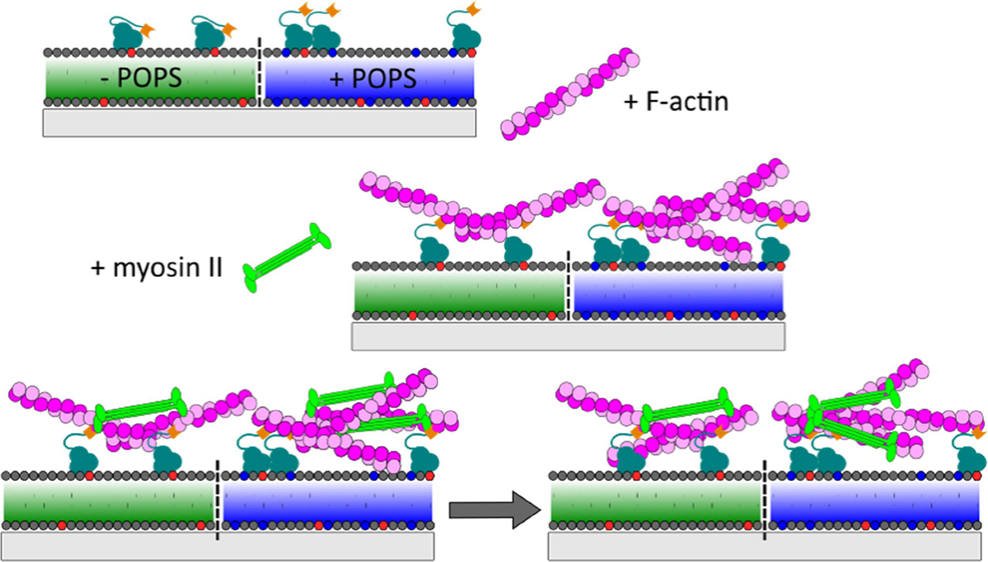

The creation of biologically inspired artificial lipid bilayers on planar supports provides a unique platform to study membrane-confined processes in a well-controlled setting. At the plasma membrane of mammalian cells, the linkage of the filamentous (F)-actin network is of pivotal importance leading to cell-specific and dynamic F-actin architectures, which are essential for the cell’s shape, mechanical resilience, and biological function. These networks are established through the coordinated action of diverse actin-binding proteins and the presence of the plasma membrane. Here, we established phosphatidylinositol-4,5-bisphosphate (PtdIns[4,5]P_2_)-doped supported planar lipid bilayers to which contractile actomyosin networks were bound via the membrane–actin linker ezrin. This membrane system, amenable to high-resolution fluorescence microscopy, enabled us to analyze the connectivity and contractility of the actomyosin network. We found that the network architecture and dynamics are not only a function of the PtdIns[4,5]P_2_ concentration but also depend on the presence of negatively charged phosphatidylserine (PS). PS drives the attached network into a regime, where low but physiologically relevant connectivity to the membrane results in strong contractility of the actomyosin network, emphasizing the importance of the lipid composition of the membrane interface.

## Introduction

Cell shape, mechanics, and dynamics predominately rely on the architecture and activity of a 100–500 nm thin actomyosin cortex located beneath the plasma membrane. In nonmuscle cells, the cortex is formed by an apolar, disordered network of transiently cross-linked actin filaments prestressed by the presence of nonprocessive myosin II motors and attached to the plasma membrane by specific anchors.^1,2^ This pre-stress gives rise to a substantial contractile tension that is essential for changing the cell’s shape during migration, maturation, and division. Cells regulate the contractility of the actomyosin cortex locally and transiently by altering the density of cross-links and motor activity. The network is attached to the plasma membrane that not only serves as a simple geometric boundary but also transmits intracellular and extracellular signals controlling specific lipid–protein interactions that dynamically influence the cytoskeletal network. Thus, membrane–cytoskeleton interactions are central to the cytoskeletal remodeling of mammalian cells.

The members of the ezrin/radixin/moesin (ERM) protein family are prototypical membrane–actin linkers.^3^ They are evolutionary highly conserved, tissue-specific, and support particular F-actin architectures in cells. For example, ERM proteins stabilize membrane protrusions^4^ such as microvilli in the brush border of the gut.^5^ In these epithelial cells, ezrin is the main player linking, with its N-terminal FERM domain (N-ERMAD) bound to phosphatidylinositol-4,5-bisphosphate (PtdIns[4,5]P_2_) in the plasma membrane,^6^ the F-actin network via its C-terminal domain (C-ERMAD).^7,8^ The recruitment of ezrin from the cytosol (inactive state) to the plasma membrane (active state) is a reversible and fine-tuned process.^9^ Activation of the inactive dormant state, in which a head-to-tail intra- (monomer) or intermolecular (dimer) interaction between the FERM domain and the C-ERMAD masks the actin-binding site, requires binding of the FERM domain to PtdIns[4,5]P_2_^10,11^ and phosphorylation of threonine-567.^11,12^ Once recruited to specific plasma membrane sites,^13^ activated ezrin serves as the linker between F-actin and the plasma membrane, whereas the myosin II motors actively reorganize the network being pivotal for cell polarity, morphogenesis, and the generation and modulation of membrane tension.^14,15^ These processes depend on the local F-actin organization^2^ as well as the membrane–cortex linkage.^16^

To disentangle the different contributions comprising the membrane composition, linker proteins, and myosin motors on the actomyosin organization and contractility, bottom-up approaches have been established based on artificial model membranes to which F-actin and actomyosin networks were attached.^17^ Besides three-dimensional model systems based on giant unilamellar vesicles (GUVs) with actin cortices attached either to the outside^18−20^ or the inside of the GUV,^19,21,22^ planar supported lipid bilayers (SLBs) turned out to be particularly suitable as they allow for high-resolution fluorescence microscopy of the F-actin network organization and dynamics. Several strategies have been pursued to link F-actin to SLBs.^16,23−27^ However, even though these artificial planar membrane systems provide some general information about the architecture and contractility of membrane-attached actomyosin cortices, none of them bound the F-actin network with a naturally occurring linkage system. This is, however important, considering its relevance for setting the time scale of the cortical flow. Murrell and coworkers either physically attached F-actin on the membrane surface with crowding agents^26,28^ or via Ni^2+^-nitrilotriacetic acid (Ni-NTA) lipids^26^ similar to the work of Köster et al.^16,23,25^ and Ganzinger et al.^24^ All authors inserted the synthetic lipid 1,2-dioleoyl-*sn-*glycero-3-[(*N*-(5-amino-1-carboxypentyl)iminodiacetic acid)succinyl] (DOGS-NTA) loaded with Ni^2+^ and formed a complex at the membrane interface with actin-binding proteins carrying a His-tag. Alternatively, Vogel et al.^27^ and Burden et al.^29^ used the classical biotin–strep(neutr)avidin approach, where altogether four binding sites of the protein are occupied either by the biotinylated lipids or the biotinylated F-actin.

Here, we created a biologically inspired artificial membrane interface on planar supports that allow us to investigate the connectivity and contractility of an actomyosin network in a bottom-up approach. In contrast to other studies, we employed the naturally occurring linker protein ezrin in the active conformation attached to its natural receptor lipid PtdIns[4,5]P_2_. We found that these seemingly subtle changes to the membrane system matter, as on- and off-rates of the protein determine the flow dynamics of the cytoskeleton on relevant time scales.^30^ Our established system enabled us to address the fundamental question of whether the presence and abundance of phosphatidylserine (PS) in the inner leaflet of the plasma membrane plays a role in the function of the F-actin cortex. This lipid is unique as it is only found in the inner leaflet of the plasma membrane, where the actin cytoskeleton is attached to the membrane. While many different kinds of physiological responses to the loss of PS-asymmetry have been investigated,^31^ its role in cortex architecture and contraction dynamics is unexplored to date. We found that the F-actin architecture and contraction dynamics of such a minimal actin cortex (MAC) strongly depend on the surface concentration of PtdIns[4,5]P_2_, which in turn defines the surface concentration of the actin-binding protein ezrin. However, more importantly, even though ezrin does not directly bind to PS, a synergistic effect of PS and PtdIns[4,5]P_2_ leads to the emergence of large-scale membrane-associated F-actin structures. At PtdIns[4,5]P_2_ surface concentrations of 1–3 mol % being in the physiologically relevant regime,^32^ we obtain F-actin networks that together with myosin II motors display stable local contraction foci but no global collapse of the network. These findings demonstrate the potential of chemically fine-tuned membrane interfaces and shed light on the biologically pressing question of how a pre-stressed network maintains mechanical homeostasis but at the same time responds dynamically as it is required for adhesion, migration, division, and tissue formation.

## Results

### Setting Up a Biologically Inspired Membrane Interface: F-Actin Linked to PtdIns[4,5]P_2_ via Ezrin

**Figure 1:**
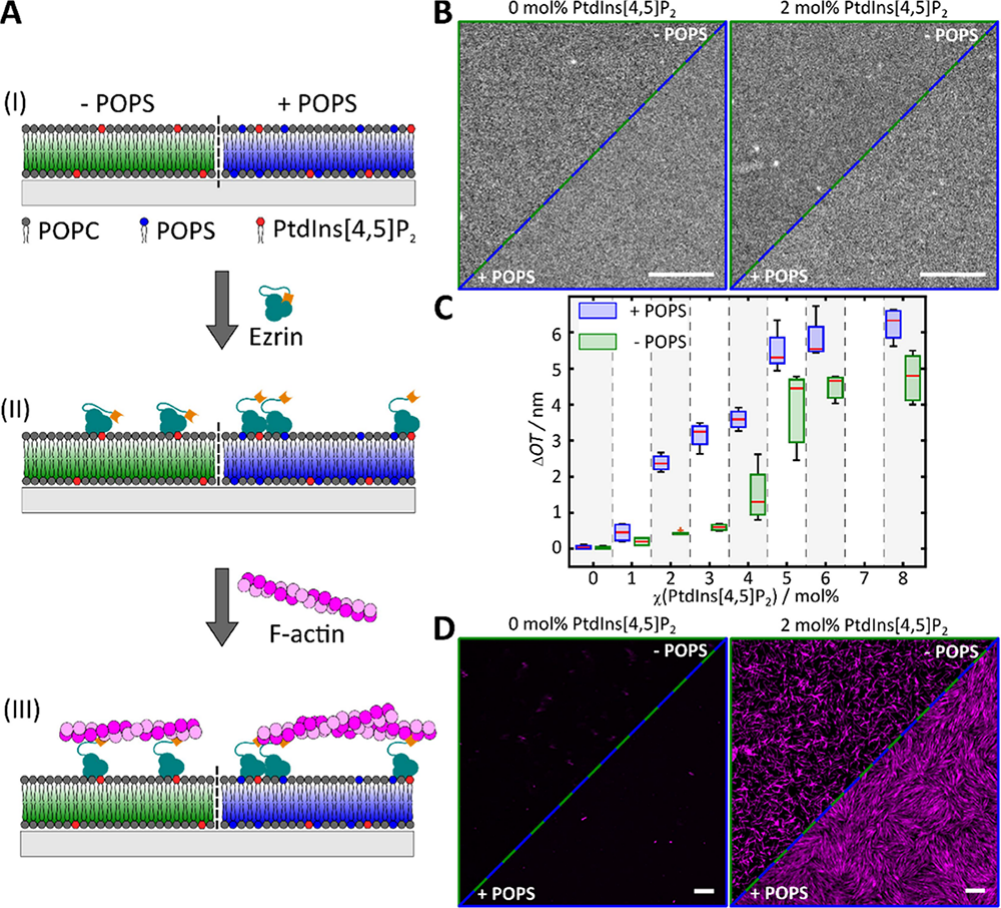
Minimal actin network attached via ezrin to SLBs. (A) Schematic illustration of the key steps of the preparation. (I) SLB formation by spreading SUVs composed of POPC/PtdIns[4,5]P_2_ (left) or POPC/PtdIns[4,5]P_2_/POPS (right) on hydrophilic surfaces. (II) Specific binding of the F-actin-membrane linker ezrin (active mutant T567D) to the receptor lipid PtdIns[4,5]P_2_ and (III) coupling of prepolymerized actin filaments. (B) Fluorescence micrographs of a glass-supported lipid bilayer composed of POPC/ATTO 390-DPPE (upper part, 99.6:0.4) and POPC/POPS/ATTO 390-DPPE (lower part, 82.6:17:0.4) (left) and POPC/PtdIns[4,5]P_2_/ATTO 390-DPPE (upper part, 97.6:2:0.4) and POPC/PtdIns[4,5]P_2_/POPS/ATTO 390-DPPE (lower part, 80.6:2:17:0.4) (right). (C) Change in optical thickness (ΔOT) caused by ezrin T567D binding to SLBs without (green) and doped with 17 mol % POPS (blue) as a function of the PtdIns[4,5]P_2_ content. For the analysis, *m* experiments were performed. 0 mol % (*m*_+PS_ = 4, *m*_-PS_= 4), 1 mol % (*m*_+PS_ = 4, *m*_-PS_ = 2), 2 mol % (*m*_+PS_ = 4, *m*_-PS_ = 5), 3 mol % (*m*_+PS_ = 4, *m*_-PS_ = 5), 4 mol % (*m*_+PS_ = 4, *m*_-PS_ = 4), 5 mol % (*m*_+PS_ = 6, *m*_-PS_ = 3), 6 mol % (*m*_+PS_ = 4, *m*_-PS_ = 3), and 8 mol % (*m*_+PS_ = 4, *m*_-PS_ = 7). ΔOT data in the absence of POPS (1–8 mol % PtdIns[4,5]P_2_) were reproduced from Nöding et al.^30^ Boxes range from 25th to 75th percentiles of the sample, while whiskers represent the most extreme data points not considered as outliers (red crosses). Medians are shown as red horizontals within the boxes. (D) Fluorescence micrographs of pre-polymerized F-actin bound to an SLB composed of POPC/ATTO 390-DPPE (upper part, 99.6:0.4) and POPC/POPS/ATTO 390-DPPE (lower part, 82.6:17:0.4) (left) and POPC/PtdIns[4,5]P_2_/ATTO 390-DPPE (upper part, 97.6:2:0.4) and POPC/PtdIns[4,5]P_2_/POPS/ATTO 390-DPPE (lower part, 80.6:2:17:0.4) (right) after incubation with ezrin T567D. Scale bars: 5 μm (B, D).

The artificial cortex was created step-by-step on the surface and each layer was analyzed by reflectometric interference spectroscopy (RIfS) and/or fluorescence microscopy. In the first step, planar supported lipid bilayers (SLBs) were produced (Figure 1A) on either glass or silicon substrates by spreading and fusing small unilamellar vesicles (SUVs).^33,34^ The SLBs were composed of 1-palmitoyl-2-oleoyl-*sn*-glycero-3-phosphocholine (POPC) and various amounts of PtdIns[4,5]P_2_. These SLBs were compared to those containing additional 1-palmitoyl-2-oleoyl-*sn*-glycero-3-phospho-l-serine (POPS). After SUV spreading, fluorescence micrographs were recorded, revealing homogeneous fluorescence verifying the formation of continuous SLBs with no domain formation (Figure 1B). The continuity of the SLBs was further confirmed by fluorescence recovery after photobleaching (FRAP) experiments. Diffusion coefficients of (3.1 ± 0.4) μm^2^/s were found, which are characteristic of lipids in SLBs^35,36^ with a small immobile fraction of (5.0 ± 2.3) % (Supporting Information, Figure S1). In a second step, the specific binding of the phospho-mimicking ezrin mutant (ezrin T567D) to the lipid bilayers was measured with the RIfS technique. This method allows quantifying protein binding without labeling the protein. Based on the interference of white light at a Si/SiO_2_ interface, a change in optical thickness ΔOT = *nd* arising from a protein (sub)layer with an average thickness *d* and a refractive index *n* is recorded, which enables one to quantify the amount of bound protein on the membrane surface.^37^ With this method, the ezrin surface coverage as a function of the PtdIns[4,5]P_2_ concentration in the bilayer became accessible. An active form of ezrin capable of binding F-actin, i.e., the ezrin T567D mutant, was used. This mutant is known to form an open conformation that binds PtdIns[4,5]P_2_ via its FERM domain and which is capable of binding F-actin via its C-terminal domain.^8,12^ To ensure full protein coverage of the PtdIns[4,5]P_2_ binding sites, an ezrin T567D concentration of 0.8 μM was chosen being in the saturation regime of the protein adsorption isotherm.^38^ The RIfS results clearly show that the protein binds with high specificity to PtdIns[4,5]P_2_. No significant ΔOT was measured after ezrin addition to pure POPC and POPC/POPS membranes, respectively (Figure 1C). However, ΔOT increases significantly if PtdIns[4,5]P_2_ was present in the membranes and was a function of the PtdIns[4,5]P_2_ surface concentration (Figure 1C).

If the PtdIns[4,5]P_2_ surface concentration χ(PtdIns[4,5]P_2_) exceeds 6 mol %, the ΔOT values reach a maximum value suggesting that the membrane surface is fully covered with protein. Previous experiments have shown that once ezrin is bound to the membrane with high surface coverage, the coverage is expected to be close to jamming since the proteins do not display mobility on the time scale of a typical FRAP experiment.^35^ Assuming a protein surface coverage close to jamming, a larger ΔOT indicates a larger protein thickness. We found the ΔOT values at χ(PtdIns[4,5]P_2_) ≥ 6 mol % to be significantly larger in the presence of POPS ((6.0 ± 0.6) nm) than in its absence ((4.7 ± 0.6) nm). From the ΔOT values, the physical thickness of the protein layer can be calculated by taking a refractive index of *n*_prot_ = 1.455^39^ into account, resulting in *d*_prot_ = (4.1 ± 0.4) nm for POPS-containing SLBs and *d*_prot_ = (3.2 ± 0.4) nm (mean ± standard deviation) for POPS-lacking SLBs, respectively.

PtdIns[4,5]P_2_ concentrations in the plasma membranes of eukaryotic cells are reported to be in the range of 1–3% of all phospholipids.^32^ In this concentration range, here given as 1–3 mol %, the ΔOT values between POPS-containing and POPS-lacking membranes are strikingly different. In this regime, we cannot distinguish between surface coverage and physical thickness of the protein layer, given by the protein’s height. Given the fact that the ΔOT values are by a factor of 2–6 larger in the presence of POPS than in its absence, this increase cannot be explained by a protein physical thickness increase alone but must be also due to a larger surface coverage.

In the third step, F-actin was bound to the membrane, in which membrane-bound ezrin served as a reversible linker between the actin filaments and the membrane.^7,30,33^ After incubation with pre-polymerized F-actin, confocal laser scanning micrographs show a quasi-two-dimensional F-actin network attached to the membrane (Figure 1D, right image). A visual inspection of the fluorescence micrographs obtained on membranes containing 2 mol % PtdIns[4,5]P_2_ with and without POPS suggests that the F-actin network is much denser in the presence of POPS (Figure 1D, right image, + POPS) than in its absence (Figure 1D, right image, – POPS). Control experiments on membranes lacking PtdIns[4,5]P_2_, which means that no ezrin has been bound, do not show any significant F-actin binding even if POPS is present (Figure 1D, left image). Also, membranes doped with PtdIns[4,5]P_2_ but in the absence of ezrin are not capable of binding F-actin (Supporting Information, Figure S2).

**Figure 2:**
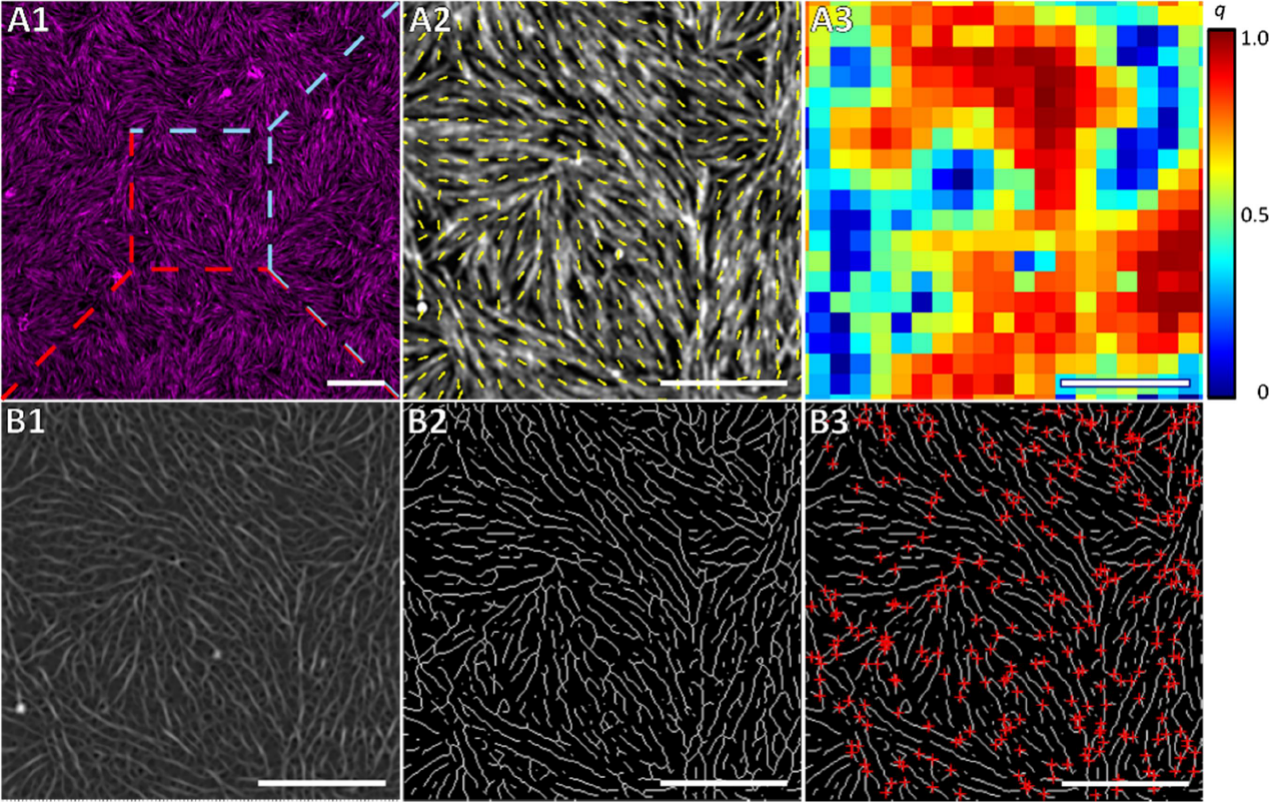
Analysis of the architecture of membrane-bound F-actin networks. (A1) Fluorescence micrograph of an F-actin network (magenta) bound to an SLB composed of POPC/PtdIns[4,5]P_2_/POPS/ATTO 390-DPPE (80.6:2:17:0.4) showing partial nematic order. (A2) Overlay of the alignment vector field (yellow arrows) and the fluorescence image (zoom in (dashed lines) of A1, shown in gray) representing the nematic phase direction within the membrane-bound F-actin network. (A3) Heat map of the scalar nematic order parameter *q* for the F-actin network shown in A1. (B1) “Tube filter” analysis of the fluorescence micrograph (zoom in (dashed lines) of A1) and (B2) the skeletonized image. (B3) Overlay of the skeletonized F-actin network with detected nodes represented as red crosses. Scale bars: 10 μm (A1) and 5 μm (A2–B3).

Besides the general increase in the amount of bound F-actin, which might be a result of the larger ezrin surface coverage at 2 mol % in the presence of POPS, we also observed that F-actin bound to POPS-containing membranes shows a different architecture, as it frequently forms local domains of the nematic alignment (Figure 1D, right image, + POPS). The nematic alignment is an indication of attractive filament–filament interactions and might simply be a result of the larger F-actin surface concentration. To be able to quantify this finding, we used an approach described by Seara et al.^40^ They determined the coarse-grained nematic order parameter *q* = 2⟨cos^2^ θ – 1/2⟩, which is a measure of the local order of the actin filaments. They found that the F-actin network continuously changes its structure from an isotropic to a nematic phase as they increase the F-actin concentration on the surface achieved by a crowding agent.^40^Figure 2A shows the evaluation procedure. Starting from the fluorescence micrograph of an F-actin network (Figure 2A1) bound to a POPS-doped SLB with an apparent nematic order, a vector field is derived representing the nematic phase direction within the membrane-bound F-actin network (Figure 2A2). From this image, the scalar nematic order parameters (*q*) are calculated, which are displayed in Figure 2A3 as a heat map. For perfectly aligned regions of the network, there is no difference in alignment, and *q* = 1, indicating perfect nematic ordering, while maximum disorder results in *q* = 0, respectively.

To correlate the F-actin network architecture given by the nematic order parameter *q* to the network density on the membrane surface, we also had to quantify the F-actin network density from the fluorescence micrographs. For this purpose, we applied a “tube filter” analysis to the fluorescence micrographs (Figure 2B1) to skeletonize the network (Figure 2B2). From the skeletonized images, we then determined the skeleton network density, defined as the ratio of pixels of the filaments (skeleton) to all image pixels. In addition, the skeletonized images enabled us to determine the node density of the networks, defined as the number of filament intersections per unit area (Figure 2B3, red crosses).^30^

### Impact of POPS and PtdIns[4,5]_2_ on the F-Actin Architecture

**Figure 3:**
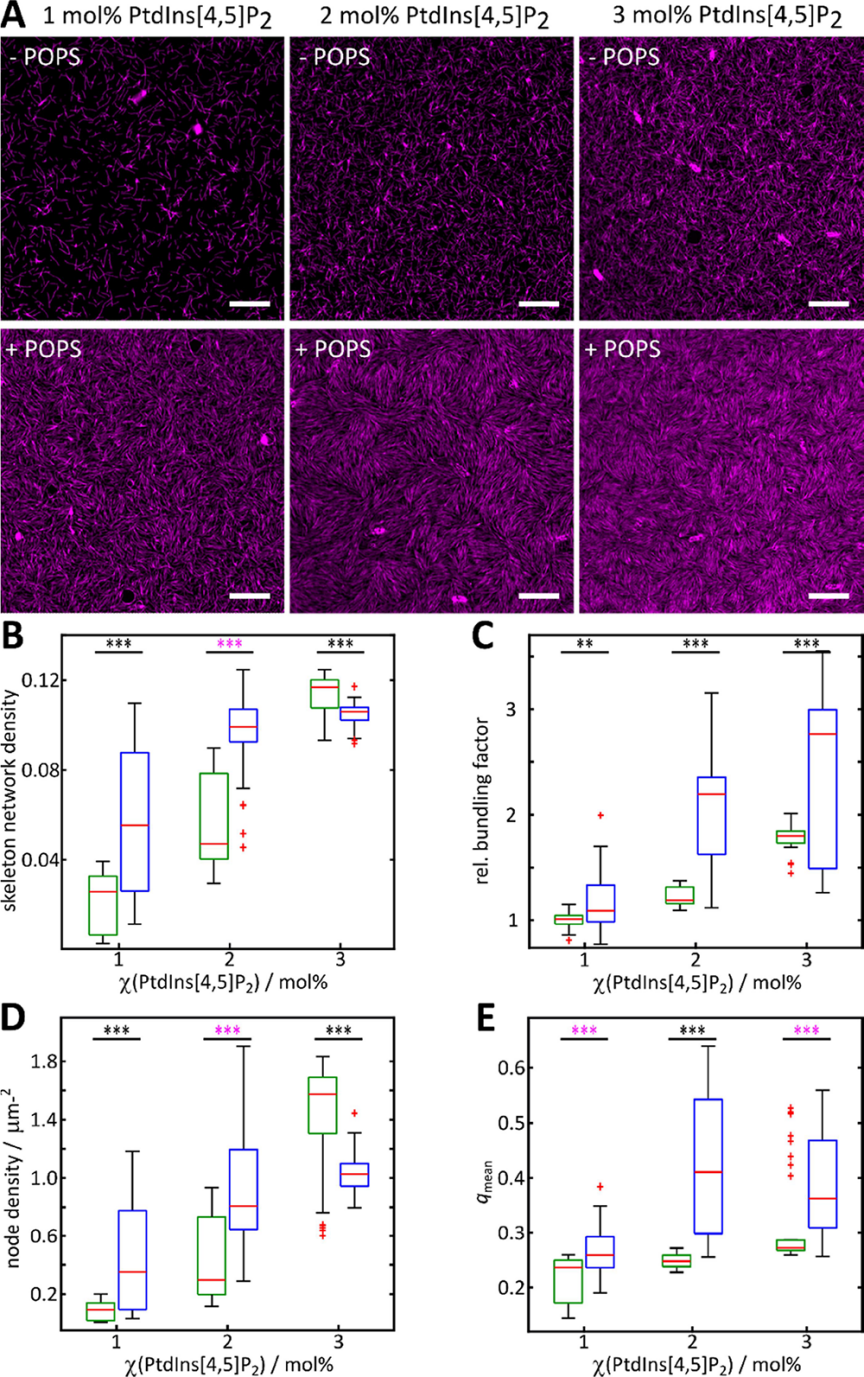
F-actin network architecture as a function of PtdIns[4,5]P_2_ and POPS content. (A) Fluorescence micrographs of membrane-bound F-actin (magenta) on SLBs lacking POPS (upper row) and doped with 17 mol % POPS (lower row) and varying PtdIns[4,5]P_2_ concentrations. Scale bars: 10 μm. (B) Skeleton network density, (C) relative bundling factor, (D) node density, and (E) mean nematic order parameter (*q*_mean_) of F-actin networks bound to SLBs without POPS (green) and doped with 17 mol % POPS (blue) as a function of the PtdIns[4,5]P_2_ content. For the analysis, *n* images of *m* independent sample preparations were evaluated. 1 mol % (*n* = 30, *n*_q,mean_ = 30, *m* = 4), 2 mol % (*n* = 23, *n*_q,mean_ = 24, *m* = 3), and 3 mol % (*n* = 34, *n*_q,mean_ = 39, *m* = 4) PtdIns[4,5]P_2_; 1 mol % (*n* = 34, *n*_q,mean_ = 37, *m* = 4), 2 mol % (*n* = 60, *n*_q,mean_ = 52, *m* = 6), and 3 mol % (*n* = 40, *n*_q,mean_ = 56, *m* = 6) PtdIns[4,5]P_2_ + 17 mol % POPS. Boxes range from 25th to 75th percentiles of the sample, while whiskers represent the most extreme data points not considered as outliers (red crosses). Medians are shown as red horizontals within the boxes. Statistical *t*-test: **: *p* ≤ 0.01, ***: *p* ≤ 0.001; Welch-test: ***: *p* ≤ 0.001.

Based on the skeletonized fluorescence images and the nematic order parameters, we were able to quantitatively investigate the influence of PtdIns[4,5]_2_ and POPS in the membrane on the F-actin structure. We first varied the PtdIns[4,5]_2_ in a concentration range of 1–3 mol % being in a typical range for the inner leaflet of mammalian plasma membranes.^32,41^ At a low PtdIns[4,5]P_2_ concentration of 1 mol %, only a few filaments bind to the ezrin-decorated membrane (Figure 3A, top left). With an increasing amount of PtdIns[4,5]P_2_, the surface concentration of actin filaments increases (Figure 3A, top middle and right), which is also reflected in the skeleton network density (Figure 3B). Whereas the network density derived from the skeletonized images nicely recapitulates single filaments in two dimensions on the membrane surface, it does not take the fluorescence intensity of the filaments into account, thus neglecting filament bundling. Hence, we arbitrarily defined a relative bundling factor being one at 1 mol % PtdIns[4,5]_2_ assuming that bundling is a rare event at the low PtdIns[4,5]_2_ concentration. The relative bundling factor increases with the PtdIns[4,5]_2_ concentration up to 1.8 at 3 mol % PtdIns[4,5]P_2_.

In the second set of experiments, we supplemented, in addition to PtdIns[4,5]_2_, 17 mol % POPS to the SLBs. In the presence of POPS, the F-actin density was found to be significantly larger at 1 and 2 mol % PtdIns[4,5]P_2_. Already at 1 mol % PtdIns[4,5]P_2_, the network is clearly visible in the fluorescence micrographs (Figure 3A, bottom left) and evolves into a dense network at 2 mol % PtdIns[4,5]P_2_ (Figure 3A, bottom middle) concomitant with an increased skeleton network density at 1 and 2 mol % PtdIns[4,5]P_2_ (Figure 3B).

At 3 mol % PtdIns[4,5]P_2_, the skeleton network density in the presence of POPS does not show an elevated network density compared to that in the absence of POPS. However, the relative bundling factor of the filaments becomes much more pronounced in the presence of POPS (Figure 3C) increasing to 2.8 ± 0.5 (median ± median absolute deviation) at 3 mol % PtdIns[4,5]P_2_ compared to 1.8 ± 0.1 for membranes in the absence of POPS (vide supra).

As long as the actin filaments are isotropically distributed on the membrane surface, also the node density is expected to increase with an increased network density (Figure 3D). Indeed, in the absence of POPS, the projected node density shows the same trend as the skeleton network density (Figure 3B) increasing from 0.09 ± 0.06 μm^–2^ (1 mol % PtdIns[4,5]P_2_) to 1.6 ± 0.2 μm^–2^ (3 mol % PtdIns[4,5]P_2_). This trend is, however, less pronounced for F-actin networks bound to POPS-containing membranes, which might be a result of the observation that the filaments arrange within the domains with nematic order. To quantify such a nematic order, we calculated the average nematic order parameter *q*_mean_ for each lipid composition (Figure 3E). For F-actin bound to SLBs lacking POPS, *q*_mean_ ranged between 0.24 ± 0.02 and 0.27 ± 0.01 reporting that no significant nematic ordering is visible on membranes harboring 1–3 mol % PtdIns[4,5]P_2_. However, *q*_mean_ calculated for networks on SLBs doped with POPS increased from 0.26 ± 0.02 at 1 mol % to 0.36 ± 0.07 at 3 mol % PtdIns[4,5]P_2_.

The most straightforward explanation for the observation that domains with nematic order are formed might be found in the packing density of the actin filaments. It was reported that the packing density of actin filaments above a critical concentration results in filament alignment.^42−44^ In our setup, the F-actin density on the membrane can be controlled by the ezrin surface coverage, which is increased with a larger PtdIns[4,5]P_2_ concentration in the membrane. Thus, to investigate whether a larger F-actin density on the membrane surface is sufficient to explain the emergence of a nematic F-actin architecture, we prepared POPC bilayers with PtdIns[4,5]P_2_ concentrations larger than 3 mol % to be able to bind more ezrin in the absence of POPS (Figure 1C). At 5 and 8 mol % PtdIns[4,5]P_2_, the ΔOT values, reporting on the ezrin surface coverage, are slightly larger than the ΔOT value at 3 mol % PtdIns[4,5]P_2_ in the presence of POPS (Figure 1C), suggesting that a similar F-actin density is bound to the ezrin-decorated membrane surface. The analysis of the fluorescence micrographs of the attached actin networks indicated (Supporting Information, Figure S3) however that the skeleton network density does not change significantly at 5 and 8 mol % PtdIns[4,5]P_2_ and remains at a value of 0.10–0.11 μm^–2^ (Supporting Information, Figure S3E), which is very similar to the value found at 3 mol % PtdIns[4,5]P_2_ in the presence of POPS. An increased F-actin density with a larger PtdIns[4,5]P_2_ content is thus not reflected in the skeleton network density. However, the relative bundling factor (Supporting Information, Figure S3F) increases with increasing PtdIns[4,5]P_2_ concentration up to 2.5 ± 0.1 (5 mol %) and 3.2 ± 0.2 (8 mol %). Hence, more actin accumulates on the membrane surface by forming bundles. To relate the F-actin density to nematic ordering, we determined *q*_mean_ for the networks on 5 and 8 mol % PtdIns[4,5]P_2_ (Supporting Information, Figure S3C) in the absence of POPS. The parameter *q*_mean_ slightly increased to 0.32 ± 0.01 (5 mol %) and 0.30 ± 0.02 (8 mol %), respectively, which agrees with the assumption that a larger density favors nematic ordering. However, the order is still lower than that in the presence of POPS, where we found 0.40 ± 0.10 for 2 mol % PtdIns[4,5]P_2_ and 0.36 ± 0.10 for 3 mol % PtdIns[4,5]P_2_ (Figure 3E).

### Myosin II-Induced Reorganization of Membrane-Bound Minimal Actin Networks

**Figure 4:**
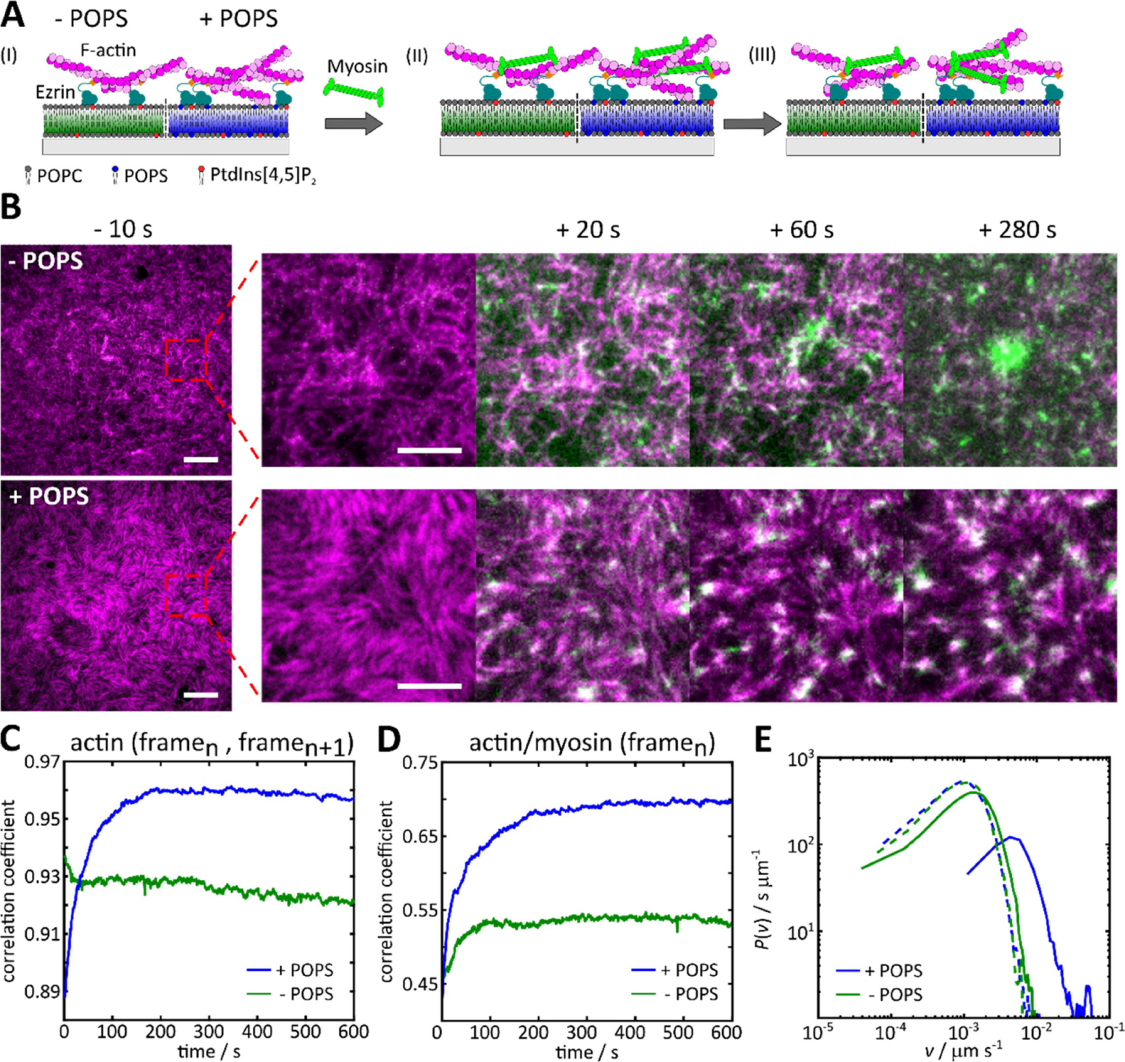
Myosin II induced reorganization of membrane-bound minimal actin networks. (A) Schematic illustration of the envisioned myosin II induced reorganization of F-actin networks bound to SLBs composed of POPC/PtdIns[4,5]P_2_ (left, green) or POPC/PtdIns[4,5]P_2_/POPS (right, blue). (I) Membrane-bound F-actin network prior to myosin II addition, (II) immediately after myosin II binding and (III) after reorganization. (B) Fluorescence micrographs of F-actin networks (magenta) bound to SLBs (χ(PtdIns[4,5]P_2_ = 3 mol %) without POPS (top row) and doped with 17 mol % POPS (bottom row) prior (−10 s) and after (+20–280 s) the initial myosin II (light green) binding (set to *t* = 0 s). Scale bars: 5 μm (left) and 10 μm. (C) Time-dependent 2D cross-correlation coefficients of the F-actin fluorescence intensity from each frame (frame_*n*_) to the following frame (frame_*n*+1_) and (D) for the F-actin fluorescence intensity to myosin II fluorescence intensity within the same frame for the images shown in B. (E) Velocity magnitude distribution derived from the images shown in B at 20 s (solid lines) and 200 s (dashed lines).

In the cortex of living cells, the architecture and connectivity of F-actin networks go hand-in-hand with the myosin II motor activity constituting a pre-stressed and contractile network capable of performing various tasks that require quick shape changes of the cell such as adhesion, migration, or division. Our artificial planar system now permits to address the question of how the organization of the F-actin network and its linkage to the membrane influences the reorganization of the network induced by the motor protein myosin II. To localize both actin filaments (magenta) and myosin motors (green), dual-color fluorescence microscopy images were acquired using total internal reflection fluorescence (TIRF) microscopy. The bipolar myosin II filaments were added to pre-assembled F-actin networks attached via ezrin T567D on PtdIns[4,5]P_2_-doped SLBs either containing or lacking POPS (Figure 4A), respectively. In the absence of POPS, myosin II binding onto the F-actin network was observed a few seconds after its addition onto the F-actin decorated SLBs (Figure 4B, top, +20 s), which started leveling off after about 20 s, as deduced from the time-dependent readout of the normalized fluorescence intensity of myosin II (Supporting Information, Figure S4B, green). Concomitant with myosin binding, the fluorescence intensity of F-actin decreased (Supporting Information, Figure S4A, green), which can be attributed to bundling, compaction, and removal of the filaments from the membrane surface.^24^ Over time, myosin II clusters formed but only a minor network reorganization was observed (Figure 4B, top, +20–280 s), i.e., large-scale reorganization of F-actin such as global or local contraction of the network does not occur (Supporting Information, Movie S1 and Figure S4A,B, blue). In contrast to the system in the absence of POPS, a large-scale F-actin reorganization was observed already after 20 s (Figure 4B, bottom, +20 s), leading to the emergence of local actin filament clusters with dense myosin centers, so-called asters (Figure 4B, bottom, +20–280 s). Rapid restructuring of the F-actin network into asters is accompanied by a loss of F-actin fluorescence intensity in their vicinity, leading to local network contraction (Supporting Information, Movie S2). In the case of SLBs containing POPS, myosin II binding occurred within the same time window.

To quantify the extent of F-actin reorganization due to myosin II activity and to quantitatively access the differences between a noncontracting and a locally contracting F-actin network, we first performed cross-correlation analyses. The actin fluorescence signal in each frame (frame_*n*_) was correlated with the actin fluorescence signal in the following frame (frame_*n*+1_) (Figure 4C). A correlation coefficient close to 1 indicates a high similarity of the frame with the following image; comparing two random images would give a correlation coefficient of 0. For the noncontracting F-actin network bound to a POPS-free membrane, the correlation coefficient drops to 0.93 as a result of minor reorganization processes such as bundling, compaction, and removal of the filaments from the membrane surface as well as fluorophore bleaching (Supporting Information, Figure S4A). In contrast, the correlation coefficient for the contracting network observed on SLBs containing POPS drops to 0.89 but increases again to 0.96 over 200 s, indicating that a more stable contracted structure is formed. Cross-correlating the F-actin and myosin II fluorescence signal in the same frame (frame_*n*_) demonstrates that during the first 100 s, where myosin II binds to the actin filaments (Supporting Information, Figure S4B), the correlation coefficient increases more in case of a locally contracting network compared to a noncontracting one (Figure 4D). These two cross-correlation coefficients could be used to distinguish between noncontracting and locally contracting networks. In addition to the two extremes of a contracting and noncontracting network, we also found cases, in which aster formation occurs only very locally (contracting), while other parts show only minor reorganization (noncontracting). These networks were classified as partially contracting networks (Supporting Information, Movie S3).

Contractility of an actomyosin network is further reflected in the velocity magnitude distribution that can be obtained from particle image velocimetry (PIV) (Figure 4E). The velocity probability distributions 20 s (solid lines) after binding of myosin II demonstrate that contractile networks show significantly larger velocities at the onset of reorganization (Figure 4E, blue solid line), while noncontractile systems remain in the lower velocity regime (Figure 4E, green solid line), similar to the situation at 200 s (Figure 4E, dashed lines), where the velocity decreases as the major reorganization has already taken place.

**Figure 5:**
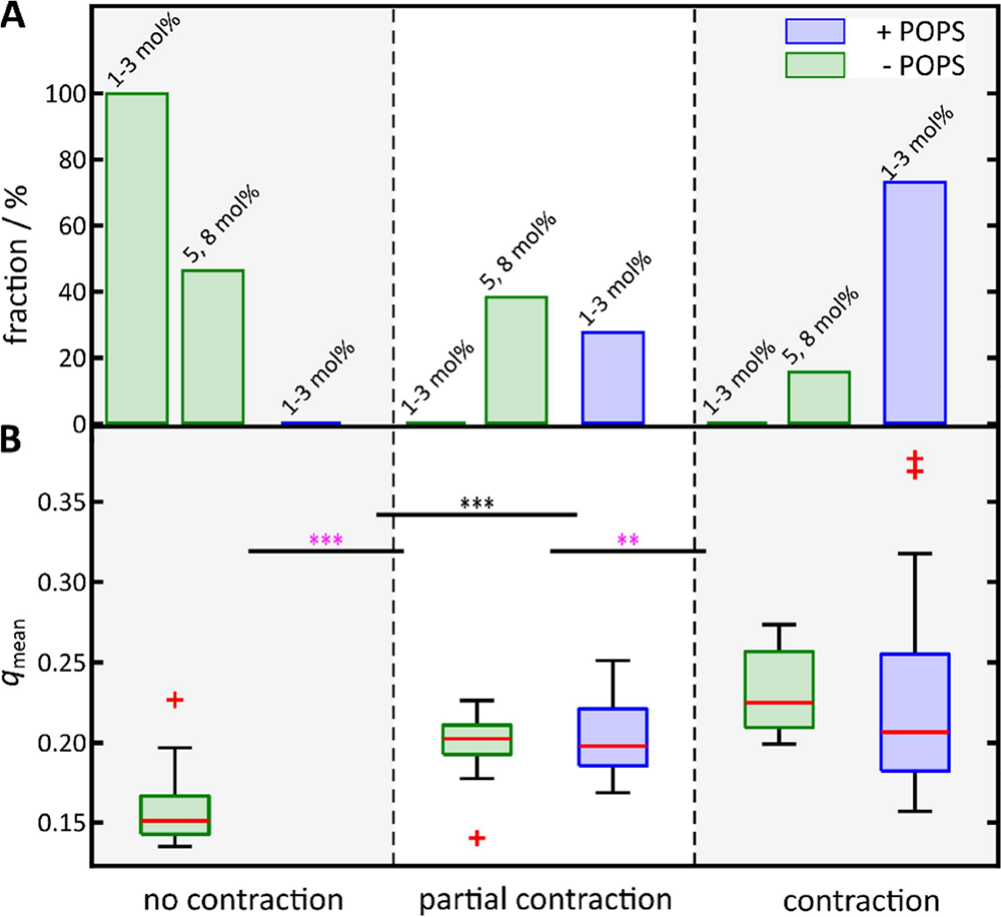
Dependence of the lipid membrane composition and mean nematic order parameter on the myosin II-induced network contractility. (A) Fraction of membrane-bound F-actin networks with a receptor lipid content of 1–3 and 5, 8 mol % PtdIns[4,5]P_2_ showing no, partial or complete network contraction upon myosin II addition as function of the POPS content. For the analysis, *n* contraction experiments were evaluated. No contraction: 1–3 mol % (*n* = 13) and 5, 8 mol % (*n* = 6) PtdIns[4,5]P_2_. Partial contraction: 5, 8 mol % (*n* = 5) PtdIns[4,5]P_2_ and 1–3 mol % (*n* = 3) PtdIns[4,5]P_2_ + 17 mol % POPS. Full contraction: 5, 8 mol % (*n* = 2) PtdIns[4,5]P_2_ and 1–3 mol % (*n* = 8) PtdIns[4,5]P_2_ + 17 mol % POPS. (B) Mean nematic order parameter (*q*_mean_) of F-actin networks bound to SLBs doped with (blue) and without (green) 17 mol % POPS as a function of the myosin II reorganization. For the analysis, *n* images of *m* preparations were used. No contraction (*n* = 55, *m* = 19), partial contraction (*n* = 20, *m* = 5; *n* = 12, *m* = 3), and full contraction (*n* = 9, *m* = 2; *n* = 33, *m* = 8). Boxes ranging from 25th to 75th percentiles of the sample, while whiskers represent the most extreme data points not considered outliers (red crosses). Medians are shown as red horizontals within the boxes. Statistical *t*-test: ***: *p* ≤ 0.001; Welch-test: ***: *p* ≤ 0.001, **: *p* ≤ 0.01.

Based on the criteria of contracting and noncontracting F-actin networks (Figure 4C,D,E), we classified each actin network either as (i) contracting, (ii) partially contracting, or (iii) noncontracting (Supporting Information, Figure S5). This enabled us to correlate the contractility of a network to the SLB lipid composition (± POPS), which influences the architecture of the network. Figure 5A shows the fraction of membrane-bound minimal F-actin networks for each classification and related to the presence of POPS in the SLBs. The results show that in the absence of POPS at PtdIns[4,5]P_2_ concentrations of 1–3 mol %, neither partial nor full local network contraction is observed. Particularly, networks at 1 mol % are unable to contract due to their intrinsically loose structure. Conversely, in the presence of POPS and at the same PtdIns[4,5]P_2_ concentrations of 1–3 mol %, only partially or even fully locally contracting networks were found. To drive the network, attached to a membrane lacking POPS, into a regime, where contraction becomes more likely, a much larger PtdIns[4,5]P_2_ concentration of 5 or even 8 mol % was required. However, even at these elevated PtdIns[4,5]P_2_ concentrations, only in about 16% of the cases, full local contraction was observed, whereas 73% of the networks showed full local contraction in the presence of POPS, suggesting that POPS alters not only the F-actin architecture on the membrane surface but also the network contractility by providing sufficient configurational freedom.

## Discussion

In recent years, a picture has emerged based on gel-based in vitro and in vivo contractility experiments that not only myosin motors but also the spatial arrangement and physical properties of the actin filament network strongly affect contractile tension in cells.^14,45−47^ Blanchoin and coworkers developed a model for the actin-dependent contractile response showing that it is a function of the connectivity following a bell-shaped curve with a maximum that is dependent on the architecture of the F-actin network.^48^ For F-actin gels, Koenderink and coworkers^49^ identified a narrow regime of motor activity and cross-linker density, in which the F-actin network displays contractile behavior. Murrell and coworkers recently showed that high concentrations of Arp2/3 result in highly branched actin networks that attenuate contractility.^50^ In nonmuscle cells, a large fraction of the F-actin network—the actin cortex—is linked to the plasma membrane, which raises the question, of how the membrane itself influences the F-actin network architecture and myosin-induced contractility of the network.

To address this question, we established a biologically inspired membrane interface, namely, planar supported lipid bilayers enabling us to control the membrane composition and interface charge as well as the F-actin anchoring using the prototypical actin–membrane linker protein ezrin. We used POPC membranes with different surface concentrations of PtdIns[4,5]P_2_ and a surface concentration of phosphatidylserine typically found in plasma membranes.^51^ Both lipids render the membrane interface negatively charged, i.e., PtdIns[4,5]P_2_ has a net negative charge of −3 to −5^52,53^ dependent on the pH value, and phosphatidylserine has a charge of −1. This is in contrast to previous studies, where the linkage of the F-actin network to the lipid membrane relied on the artificial lipid DOGS-NTA(Ni^2+^)^16,23−26^ or even on positively charged lipids.^54^ Electrostatics, repulsive or attractive, are, however, not the only parameter to be considered for F-actin to interact with a planar membrane. On neutral POPC membranes, also no actin filaments became discernable (Figure 1D). This finding is a consequence of the shear presence of a nondeformable (membrane) surface resulting in depletion forces that arise due to the substantial persistence lengths of the actin filaments and considerably reduce the concentration of F-actin close to a wall or membrane.^55^ This aspect emphasizes the need for firm F-actin attachment sites on the membrane as realized by proteins of the ERM family. However, a balance between tight binding and configurational freedom must be maintained to allow cortical flow on reasonable time scales.

Ezrin serves as the linker between the membrane and F-actin. It specifically binds to PtdIns[4,5]P_2_ containing SLBs, but not to POPC or POPS (Figure 1C). However, the change in optical thickness (ΔOT) upon protein binding is significantly larger in the presence of POPS than in its absence at the same PtdIns[4,5]P_2_ concentration. To interpret this finding, two contributions need to be considered, namely, the protein surface coverage and its height, i.e., the thickness of the pure protein layer. The thickness of the protein layer can be obtained at a protein surface coverage close to the jamming limit. This is achieved at χ(PtdIns[4,5]P_2_) ≥ 6 mol % indicated by ΔOT values that reached a maximum. In this receptor concentration regime, the thickness of the ezrin layer is with *d*_prot_ = (4.1 ± 0.4) nm (+ POPS) about 30% larger than in the absence of PS (*d*_prot_ = (3.2 ± 0.4) nm, – POPS). The latter value is in good agreement with the expected height of a densely packed membrane-bound ezrin layer on PtdIns[4,5]P_2_/DPPC bilayers ((3.0 ± 0.4) nm) as determined by atomic force micrographs.^11^ It is conceivable that POPS itself increases the height of ezrin due to an altered protein organization on the membrane surface. The α-helical linker region and the C-terminal C-ERMAD^8^ exhibit a negative net charge (estimated via Protpi.ch) suggesting repulsive interactions with the negatively charged membrane, which might lead to an altered, more elongated ezrin structure. This is, however, not the only contribution to the observed protein height *d*_prot_, as a mere addition of negative charges in the membrane by replacing PS with phosphatidylglycerol (PG) does not lead to a larger *d*_prot_ (Supporting Information, Figure S6).

At 1–3 mol % PtdIns[4,5]P_2_ the interpretation of the observed larger ΔOT values is less straightforward since both the protein height and coverage need to be considered, the latter being determined by the number of PtdIns[4,5]P_2_ binding sites. An increase in the protein height of about 30% can obviously not explain the observed large ΔOT values in the presence of POPS. It is more likely that, even though ezrin does not bind to PS itself, an enhanced amount of protein binds in the presence of PS owing to a synergistic effect of the negative charges.^56^ Indeed, if PS is replaced by PG, the ΔOT values are again larger than those in the absence of negatively charged lipids (Supporting Information, Figure S6).

These results demonstrate that already a subtle change in membrane composition (+ POPS) influences the protein linker-decorated membrane surface and thus can affect the linkage between the membrane and the actin filaments. Indeed, at 1–3 mol % PtdIns[4,5]P_2_ and in the presence of POPS, the F-actin density is significantly increased concomitantly with a network with nematic order. According to Onsager^44^ and the extended model of Khokhlov and Semenov^43^ for semiflexible rods like F-actin, the formation of nematically aligned filaments depends on the critical packing density at which filaments align and thereby reduce their excluded volume.^42^ Critical actin concentrations in the solution, leading to nematic phases, can range between 75–100 μm^57^ and can go down to 2.3–5 μm if crowding agents are applied.^40^ We observed the transition from an isotropically to a nematically organized network at an actin concentration of only 1.7 μm in the absence of crowding agents, suggesting that the local packing of F-actin in a quasi-two-dimensional arrangement on the membrane readily leads to a density that is above the critical concentration required for filament alignment. However, only increasing the F-actin density on the membrane surface by increasing the PtdIns[4,5]P_2_ concentration, i.e., the ezrin surface coverage similar to that found in the presence of POPS, does not fully recover the nematic ordered architecture of the F-actin network found in the presence of POPS (Supporting Information, Figure S3). Therefore, we presume that the negatively charged lipid POPS itself in combination with a more elongated ezrin conformation also contributes to the F-actin structure on the membrane surface. F-actin depletion at a planar surface is expected to be less pronounced if bound at a larger distance between the membrane and the actin network as a result of the altered ezrin conformation.^55^ Moreover, upon linkage of the actin filaments to the membrane, a secondary attractive interaction between the negatively charged PS areas in the membrane^58^ and F-actin in the presence of Mg^2+^ that screen the negative net charges^59^ is conceivable, which would increase the F-actin concentration on the membrane surface.

**Figure 6:**
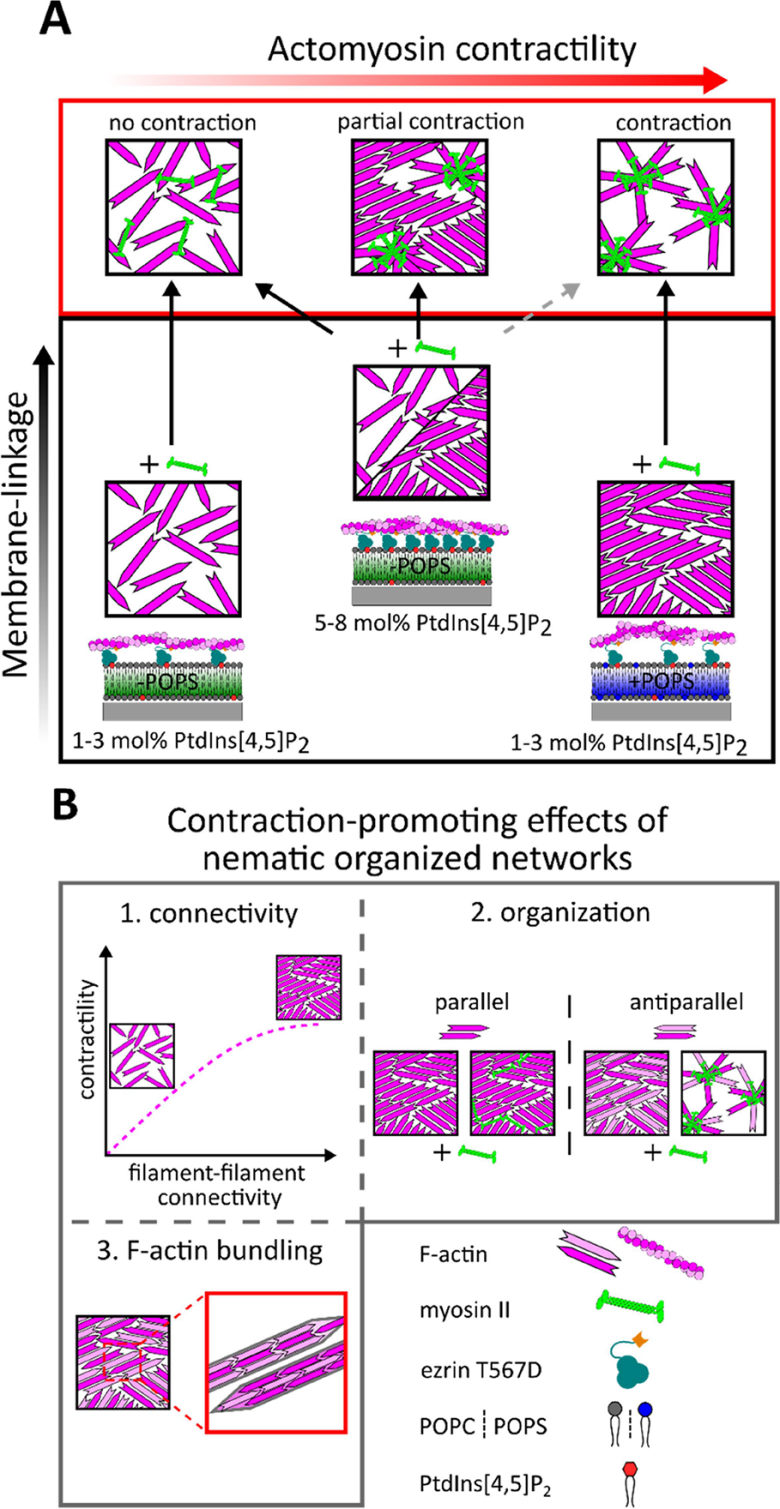
Influence of phosphatidylserine on the filament–filament connectivity and contractility of minimal actomyosin networks. (A) Local contractility is not observed in actomyosin networks attached to membranes with a low number of membrane linkages (1–3 mol % PtdIns[4,5]P_2_) and in the absence of POPS (− POPS) owing to a network architecture with low filament–filament connectivity. Only by considerably increasing the number of membrane linkages (5–8 mol % PtdIns[4,5]P_2_), the F-actin network architecture is driven into partly aligned network structures that allow partial contraction, whereas the strong connectivity to the membrane acts against contraction. In contrast, in the presence of POPS (+ POPS), a low number of membrane linkages (1–3 mol % PtdIns[4,5]P_2_) is sufficient to generate strongly aligned F-actin networks (nematic order), which contract upon myosin II action. (B) Alignment of filaments leading to an increased (1) filament–filament connectivity, (2) antiparallel organization of the filaments, and (3) F-actin bundling contributes to the enhanced contraction propensity.

Another observation is that network contraction upon the addition of myosin II motors is favored in the presence of POPS and low PtdIns[4,5]P_2_ concentrations (Figures 5A and 6). One parameter is the presence of negatively charged POPS in conjunction with the positioning of ezrin fulfilling the task of a (sliding) boundary that provides the required configurational freedom for the actin filaments.^60,61^ The other parameter is filament alignment. If local contraction were only controlled by the skeleton network density of the actin filaments, the contraction would also be expected at 2 and 3 mol % PtdIns[4,5]P_2_ in the absence of POPS (Figure 3B). Even if the PtdIns[4,5]P_2_ concentration is increased to 5 and 8 mol % to compensate for the absence of POPS, only a fraction of the network contracts (Figures 5A and 6).

We attribute this general ability of the actomyosin network to contract even in the presence of a strong membrane attachment to the slip bond and sliding behavior of the F-actin–ezrin interaction^7,62^ as the binding lifetime decreases with applied force. Nöding et al.^30^ found in microrheology experiments off-rate of the actin (network)–ezrin bond on the order of 0.1 s^–1^ indicative of a highly dynamic bond. However, contraction is still largely impaired showing that simply increasing the anchoring points (ezrin-PtdIns[4,5]P_2_) is not sufficient to generate local contraction.

This is in agreement with the observations reported by Murrell and Gardel,^63^ who found that the attachment of a contractile actomyosin network to a bilayer containing >8 mol % of FimA2 anchors attached via a His-tag compromises the ability of the network to form contraction foci, in contrast to a network that is only crowded onto the membrane with methyl-cellulose.

It is the enhanced filament–filament connectivity, visible as membrane-bound F-actin aligning in nematic domains and bundled filaments, that enhances the contractility and not the overall connectivity to the membrane, which restricts the configurational freedom of the filaments. It has been shown that the nematic order can increase local contraction. Contraction in nonsarcomeric structures is especially large in the nematic phase with dense two-dimensional F-actin bundles with apolar orientation,^63^ while bundled filaments enhance the contraction length scale.^64^ Blanchoin and coworkers^48^ showed that antiparallel bundles of F-actin display substantially faster contraction compared to nonordered actin meshes using in vitro contractility assays. Recently, Murrell and coworkers^50^ reported that Arp2/3-derived branched networks attached on a membrane surface even suppress network contraction due to impaired rotational and translational motion of myosin thick filaments. In contrast, formin-nucleated (mDia1) actin networks (bundles) show a clear signature of myosin-based contractility. In our system, we expect a random orientation of the individual membrane-bound actin filaments and bundles in line with the observed contraction of aligned filaments.

## Conclusions

We established a biologically inspired membrane interface with a planar geometry amenable to high-resolution fluorescence microscopy. This setup enables us to control the lipid composition and charge of the supported bilayers, which turned out to be a crucial determinant not only for the mere actin anchorage but also for the architecture of membrane-bound F-actin networks that greatly influence the contractility of an actomyosin network. Conditions of high network densities at low specific actin-binding sites and a large order of F-actin can be achieved by a fine-tuned lipid bilayer composition enabling connectivity and contractility in otherwise too-diluted networks. Given the concentrations of PtdIns[4,5]P_2_ and phosphatidylserine in the plasma membrane, they appear to be ideally chosen to meet the requirements for F-actin network connectivity and contractility in mammalian cells.

## Experimental Section

### Vesicle Preparation

Stock solutions (1–10 mg/mL) of 1-palmitoyl-2-oleoyl-*sn-*glycero-3-phosphocholine (POPC), 1-palmitoyl-2-oleoyl-*sn-*glycero-3-phospho-l-serine (POPS, Avanti Polar Lipids, Alabaster, AL, USA), and ATTO 390-1,2-dipalmitoyl-*sn-*glycero-3-phosphoethanolamine (Atto 390-DPPE, ATTO-TEC, Siegen, Germany) were prepared in chloroform. l-α-Phosphatidylinositol-4,5-bisphosphate (PtdIns[4,5]P_2_, brain porcine, Avanti Polar Lipids, Alabaster, AL, USA) was freshly dissolved in chloroform/methanol/H_2_O (10:20:8) to a final concentration of 1 mg/mL. Lipid mixtures (0.4 mg) were prepared in chloroform/methanol (10:1), and organic solvents were evaporated with a nitrogen stream followed by 3 h in vacuum. The dried lipid films were stored at 4 °C until needed.

Small unilamellar vesicles (SUVs) were prepared by rehydrating a lipid film in spreading buffer (50 mM KCl, 20 mM Na-citrate, 0.1 mM NaN_3_, 0.1 mM ethylenediaminetetraacetic acid (EDTA), pH 4.8),^38^ incubating for 30 min, subsequent vortexing (3 × 30 s at 5 min intervals), and a final sonification step for 30 min at room temperature (cycle 4, 60%, Sonopuls HD2070, resonator cup; Bandelin, Berlin, Germany). PtdIns[4,5]P_2_ containing SUVs were used immediately for the preparation of SLBs to avoid PtdIns[4,5]P_2_ degradation.^65^

### SLB Preparation

Supported lipid bilayers (SLBs) were prepared on glass substrates (no. 1.5, Marienfeld-Superio, Lauda-Königshofen, Germany), used for fluorescence microscopy imaging, and on silicon wafers coated with 5 μm SiO_2_ (Silicon Materials, Kaufering, Germany), used for reflectometric interference spectroscopy (RIfS). Both substrates were treated for 20 min with a H_2_O/NH_3_/H_2_O_2_ (5:1:1, v/v) solution at 70 °C and subsequently activated for 30 s with O_2_-plasma (Zepto LF PC, Diener electronic, Ebhausen, Germany). The hydrophilized substrates were mounted in a measuring chamber and immediately incubated with SUVs.

For the preparations on glass slides, SLBs were formed by incubating the substrates for 1 h with SUVs (*m* = 0.2 mg, *c* = 0.53 mg/mL) at 20 °C and excess lipid material was removed by a 10-fold buffer exchange with spreading buffer followed by ezrin buffer (50 mM KCl, 20 mM Tris, 0.1 mM NaN_3_, 0.1 mM EDTA, pH 7.4). For SLB formation on silicon substrates, SUVs (*m* = 0.2 mg, *c* = 0.53 mg/mL) were spread while the optical thickness was read out. After successful SLB formation, excess lipid material was removed by rinsing 5 min with spreading buffer and 5 min with ezrin buffer.

### Ezrin Binding Monitored by RIfS

Ezrin T567D was recombinantly expressed in *E. coli* (BL21(DE3)pLysS, Novagen, Madison, WI, USA) and purified as described previously.^33^ RIfS was used to measure the formation of SLBs on the silicon wafers and binding of the protein onto the membranes. RIfS is a noninvasive label-free technique to determine optical layer thicknesses (OT = *nd*). OT values were monitored using a flame-S-UV/vis spectrometer (Ocean Optics, Dunedin, FL, USA), recording a spectrum every 2 s and analyzed utilizing a custom MATLAB script (R2021a, Mathworks). The experimental setup was described previously.^37^ After SLB formation, the membrane surface was rinsed with ezrin buffer and a BSA solution (1 mg/mL in ezrin buffer) for 5 min. After rinsing again with ezrin buffer for 5 min, ezrin T567D was added (0.8 μM) for 10 min. Unbound protein was removed by rinsing with ezrin buffer.

### Preparation of Membrane-Bound F-Actin Networks

Ezrin T567D was bound to the SLBs at a concentration of 1 μm overnight at 4 °C. Excess protein was removed by a 10-fold buffer exchange with ezrin buffer and F-actin buffer (50 mM KCl, 20 mM Tris, 2 mM MgCl_2_, 0.1 mM NaN_3_, pH 7.4). For F-actin pre-polymerization, ATTO 594-NHS ester (ATTO-TEC, Siegen, Germany) labeled nonmuscle G-actin and unlabeled monomers (Cytoskeleton, Denver, CO, USA) were solved in a 1:10 ratio and a final concentration of 0.44 mg/mL in G-buffer (5 mM Tris, 0.2 mM CaCl_2_, 0.1 mM NaN_3_, pH 8.0). Actin oligomers were depolymerized by the addition of dithiothreitol (DTT, 0.5 mM) and adenosine 5′-triphosphate (ATP, 0.2 mM) for 1 h on ice. Remaining actin aggregates were centrifuged (17,000 × *g*, 20 min, 4 °C) and polymerization was induced by the addition of 10% of the total volume of polymerization solution (500 mM KCl, 20 mm MgCl_2_, 20 mM ATP, 50 mM guanidine carbonate, pH 7.4). After a polymerization time of 20 min at 20 °C, the F-actin solution was mixed with unlabeled phalloidin in a 1.5% (*n*/*n*) ratio and incubated for another 20 min. Minimal actin networks were formed at 20 °C by incubating the ezrin T567D-decorated SLBs with polymerized F-actin at a concentration of 4.6 μM for at least 2 h. Unbound filaments were washed off by a 10-fold buffer exchange with F-actin buffer.

### Contraction Experiments

Myosin II was purified from rabbit skeletal muscle and fluorescently labeled with DyeLight 488 (Invitrogen, Carlsbad, CA, USA) according to Alvarado and Koenderink.^66^ Labeled and unlabeled myosin II were stored separately in myosin storage buffer (300 mM KCl, 25 mM KH_2_PO_4_, 0.5 mM DTT, 50% (v/v) glycerol, pH 6.5), where the high ionic strength prevents myosin self-assembly into bipolar filaments. For experiments, myosin II was dialyzed overnight in glycerol-free myosin buffer (300 mM KCl, 20 mM imidazol, 4 mM MgCl_2_, 1 mM DTT, pH 7.4) and controlled self-assembly into bipolar filaments was induced by adjusting a KCl concentration of 50 mM via mixing with myosin polymerization buffer (20 mM imidazol, 1.6 mM MgCl_2_, 1 mM DTT, 1.2 mM Trolox, pH 7.4). After an incubation time of 10 min at 20 °C, the bipolar myosin II filaments were immediately used for contractile experiments. For the contraction experiments, the F-actin networks were transferred into an actomyosin buffer by a 10-fold buffer exchange (50 mM KCl, 20 mM imidazol, 2 mM MgCl_2_, 1 mM DTT, 1 mM Trolox, pH 7.4). The reorganization of the networks was performed at a final ATP concentration of 0.1 mM combined with an ATP-regeneration system of creatine phosphate (10 mM)/creatine kinase (0.1 mg/mL)^66^ and a myosin II concentration of 0.4 μM.

### Fluorescence Recovery after Photobleaching Experiments

FRAP experiments were conducted with a FluoView 1200 CLSM (Olympus, Tokyo, Japan) by recording the fluorescence intensity of SLBs doped with ATTO 488-DPPE and bleached via a rapid laser pulse (λ_bleach_ = 488 nm, 20 mW). The fluorescence intensity was tracked over time in a region of interest (Supporting Information, Figure S1A,B) and a frame time of 65 ms. The diffusion coefficient and immobile fraction calculation were performed according to Jönsson et al.^67^

### Image Acquisition

Confocal fluorescence images were acquired with the upright confocal laser scanning microscope LSM 880 (CLSM, Carl Zeiss Microscopy GmbH, Oberkochen, Germany) using a 40× objective (W Plan-Apochromat M27, NA = 1.0, Carl Zeiss Microscopy GmbH, Oberkochen, Germany). The membrane dye ATT0 390-DPPE was excited at λ_ex_ = 405 nm (diode laser, 30 mW) and detected at 450–550 nm. The ATTO 594-labeled F-actin was excited at λ_ex_ = 561 nm (diode laser, 20 mW) and fluorescence was detected at 600–700 nm, using an Airyscan detector.

Dual color TIRF microscopy was performed using the IXpolre TIRF system (cellTIRF-4Line, Olympus Deutschland GmbH, Hamburg, Germany) equipped with a 100× oil objective (UPLAPO-HR, NA = 1.5, Olympus Deutschland GmbH, Hamburg, Germany). The ATTO 594-labeled F-actin was excited at λ_ex_ = 561 nm (diode laser, 100 mW) and DyLight 488-labeled myosin II at λ_ex_ = 488/491 nm (diode laser, 500/100 mW). Exposure times were 20 ms (myosin II) and 40 ms (actin), and fluorescence signals were detected using a Zyla 4.2 sCMOS (Andor Technology Ltd., Belfast, UK).

### Image Analysis

Fluorescence micrographs of membrane-bound F-actin networks were skeletonized by means of the Jupyter notebook based custom written “tube filter” analysis (https://github.com/AKSteinem/tubefilter). In the first step of the analysis, a contrast limited adaptive histogram equalization (CLAHE)^68^ and a two-dimensional Gaussian-filter were used to equilibrate the global image intensity, improve the local image contrast, and reduce high-frequency noise. Based on the F-actin intensity, these two-dimensional exposure-corrected micrographs (*I*(*x*, *y*)) can be described as three-dimensional surfaces. In the second analysis step, the Hessian image matrix (*H*_I_(*x*, *y*)), eq 1) of the exposure-corrected micrographs was calculated, containing the second partial derivatives of the input image:
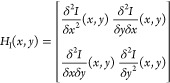
1

The signal-to-noise enhanced tube-filtered images (Figure 2B1) were generated by means of the second eigenvalues λ_2_(*x*, *y*) of *H*_I_(*x*, *y*), describing the minimal surface curvature at each image pixel of *I*(*x*, *y*). Tube-filtered images were subsequently suited for “conventional” adaptive thresholding and skeletonization.

From the skeletonized images (Figure 2B2), the skeleton network density, defined as the ratio of filamentous pixels (Figure 2B2, white) to all image pixels and filament intersections, referred to as node density (Figure 2B3, red crosses), were determined. A node was defined as a filamentous pixel with more than two adjacent filamentous pixels.

The relative bundling factor calculation was performed via extracting the fluorescence intensity of membrane-bound F-actin, by masking actin fluorescence images with the corresponding skeletonized images (Supporting Information, Figure S7). The actin fluorescence was determined at the overlapping positions, averaged for the respective micrograph, and normalized with the mean F-actin intensity at χ(PtdIns[4,5]P_2_) = 1 mol % without POPS, assuming only single actin filaments at these conditions.

The local nematic order parameter *q* = 2⟨cos^2^ θ – 1/2⟩ of membrane-bound networks was calculated according to Seara et al.^40^ by utilizing the published MATLAB routines with a 5 × 5 kernel for adjacent windows. For each fluorescence image, the mean nematic order parameter (*q*_mean_) was calculated by averaging all local *q*-values. Since the window size for the generation of the alignment vector field (Figure 2A2, yellow) significantly influences the calculated *q*-values, *q*_mean_ was computed for each image as a function of the window size in order to determine an optimal one (Supporting Information, Figure S8). The optimal window size used in this work ranged between 1–2 μm.

Actin and myosin II intensities were analyzed after the initial binding of myosin II. The respective sample intensity was averaged for each frame and normalized by means of the maximal intensity within the particular time series. Intensity correlations within one frame (frame_*n*_) or to the following frame (frame_*n*+1_) were performed for the entire time series utilizing the MATLAB function corr2 (Figure 4C,D).

For the calculation of the F-actin velocity magnitude distribution of contractile actomyosin networks, the samples were analyzed by means of particle image velocimetry (PIV) using the MATLAB-based PIVlab (version 2.53) from Thielicke and Sonntag.^69^ To compensate for thermal drift, the time series were cropped to the region of interest and subsequently adjusted using the StackReg (http://bigwww.epfl.ch/thevenaz/stackreg/) plugin of ImageJ (https://imagej.nih.gov/ij/). The PIV analysis was performed at a time difference of 10 s between the analyzed frames, using the parameters listed in the Supporting Information (Table S1). The final F-actin velocity magnitude distribution calculation was performed by averaging the F-actin velocity magnitude per frame and utilizing the MATLAB function ksdensity (Figure 4E and Supporting Information, Figure S5).

General data analysis and plotting of graphs were done using MATLAB and custom-written scripts. Basic image processing was done with ImageJ. All custom-written scripts are provided upon reasonable request.

## Supporting Information Available

FRAP experiments on supported bilayers; control experiments of ezrin binding; fluorescence micrographs of F-actin networks on membranes; time-dependent fluorescence intensity changes upon myosin II addition; mean velocity magnitude distributions; RIfS results of ezrin T567D binding as a function of the negatively charged lipids POPS and POPG; relative bundling factor determination; determination of the optimal window size for the nematic order parameter; and PIV settings for the calculation of the F-actin velocity magnitude (PDF)
Movie of a noncontractile F-actin network (MP4)
Movie of a contractile F-actin network (MP4)
Movie of a partially contracting F-actin network (MP4)
