# Supplementary material for: Bioinspired Membrane Interfaces: Controlling Actomyosin Architecture and Contractility
Source: ACS Appl Mater Interfaces. 2023 Feb 27;15(9):11586–98. doi: 10.1021/acsami.3c00061 (PMC9999349; doi:10.1021/acsami.3c00061)
Supplement: Supplementary file 1 — am3c00061_si_001.pdf [file am3c00061_si_001.pdf]

Supporting Information

Bioinspired Membrane Interfaces: Controlling Actomyosin Architecture and Contractility

Nils L. Liebe,[†] *Ingo Mey,*[†] *Loan Vuong,*[†] *Fadi Shikho,*[†] *Burkhard Geil,*[#]
Andreas Janshoff,[#] *Claudia Steinem*[†,$,*]

[†] Georg-August Universität, Institut für Organische und Biomolekulare Chemie, Tammannstr. 2, 37077 Göttingen, Germany

[#] Georg-August Universität, Institut für Physikalische Chemie, Tammannstr. 6, 37077 Göttingen, Germany

[$] Max-Planck-Institut für Dynamik und Selbstorganisation, Am Fassberg 17, 37077 Göttingen, Germany

Corresponding author: csteine@gwdg.de

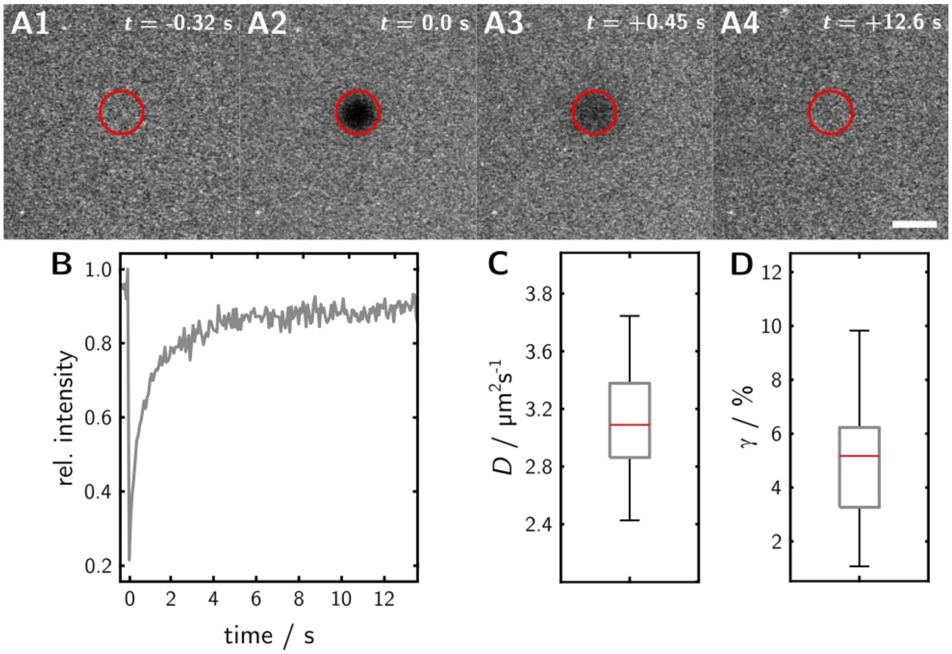

Figure S1. Lipid diffusion in solid-supported bilayers. (**A**) Time-lapse fluorescence images before (**A1**) and after bleaching (**A2**, t = 0 s) the fluorophore and the observed recovery (**A3**, **A4**) during a FRAP experiment on an SLB (POPC/PtdIns[4,5]P_2/ATTO 488-DPPE, 96.6:3:0.4). The fluorescence intensity is read out in the region of interest (ROI, red circle). Scale bar: 5 µm. (**B**) Intensity profile of a FRAP experiment. Box plot diagrams displaying the (**C**) diffusion coefficients D as well as the (**D**) immobile fractions γ of the SLBs (n = 34). The median values are indicated as red lines with $D = 3.1 \pm 0.4$ µm^{2}s^{-1} and $\gamma = 5.0 \pm 2.3$ %. Boxes range from 25th to 75th percentiles of the sample, while whiskers represent the most extreme data points.

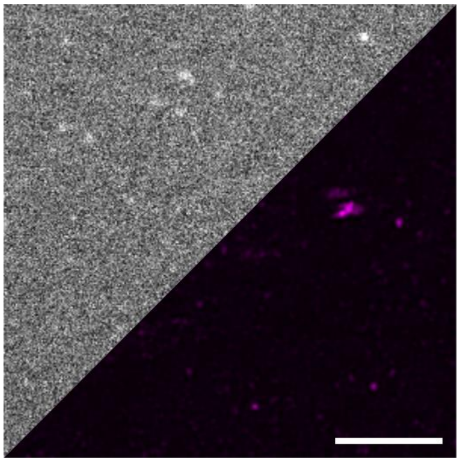

Figure S2. PtdIns[4,5]P_2-containing membranes do not bind F-actin in absence of ezrin T567D. Exemplary fluorescence micrographs of a glass-supported lipid bilayer (gray) composed of POPC/PtdIns[4,5]P_2/ATTO 390-DPPE, 91.6:8:0.4 (upper part) showing no unspecific attachment of pre-polymerized F-actin (magenta, lower part) in the absence of ezrin T567D. Scale bar: 5 µm.

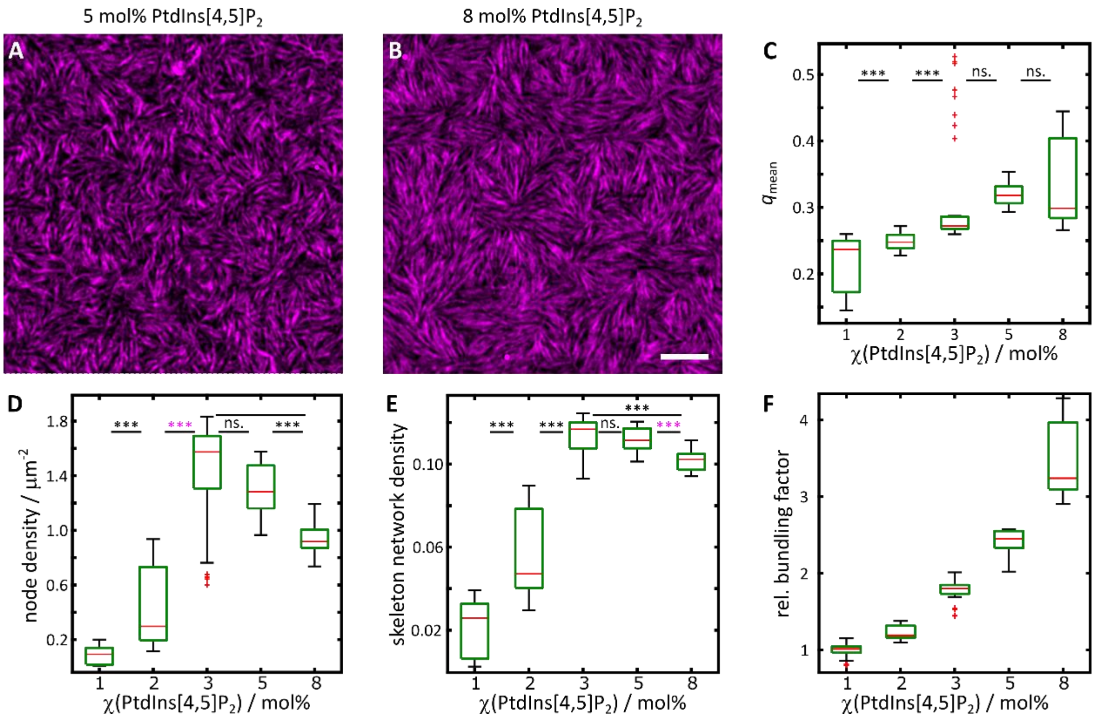

Figure S3. Comparison of the F-actin network self-organization on membranes as a function of PtdIns[4,5]P_2 content. Fluorescent micrographs of F-actin (magenta) on SLBs doped with 5 mol% (**A**) and 8 mol% (**B**) PtdIns[4,5]P_2 in the absence of POPS. Scale bar: 5 µm. (**C**) Mean nematic order parameter (q_{mean}), (**D**) node density, (**E**) skeleton network density, and (**F**) relative bundling factor of F-actin networks bound to SLBs without POPS as a function of PtdIns[4,5]P_2 content. For the analysis, n images of m preparations were evaluated. 1 mol% ($n = 30$, $n_{q,mean} = 30$, $m = 4$), 2 mol% ($n = 23$, $n_{q,mean} = 24$, $m = 3$), 3 mol% ($n = 34$, $n_{q,mean} = 39$, $m = 4$), 5 mol% ($n = 20$, $n_{q,mean} = 20$, $m = 2$) and 8 mol% ($n = 31$, $n_{q,mean} = 31$, $m = 3$) PtdIns[4,5]P_2. Boxes ranging from 25th to 75th percentiles of the sample, while whiskers represent the most extreme data points not considered as outliers (red crosses). Medians are shown as red horizontals within the boxes. Statistical t-test: ns.: $p > 0.05$, ***: $p \leq 0.001$; *Welch*-test: ***: $p \leq 0.001$.

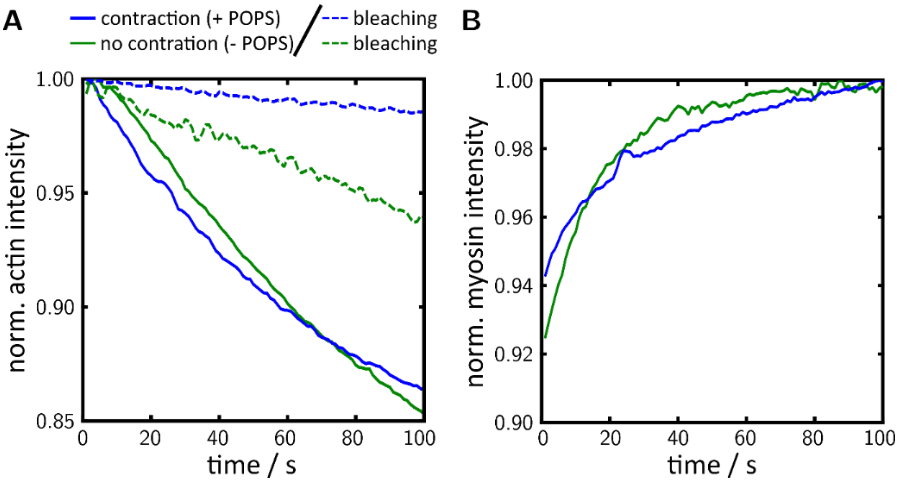

Figure S4. Time-dependent fluorescence intensity changes upon myosin II addition. Time-dependent normalized fluorescence intensities of F-actin (**A**, solid lines) and myosin II (**B**) bound to SLBs (χ(PtdIns[4,5]P_2 = 3 mol%) without POPS (green, no contraction) and doped with 17 mol% POPS (blue, contraction) after the initial binding of myosin II (set to $t = 0$ s). The dashed lines show the general bleaching of the F-actin fluorescence in the absence of myosin (A, dashed lines). Time-dependent intensities were extracted from the time series shown in Figure 4B.

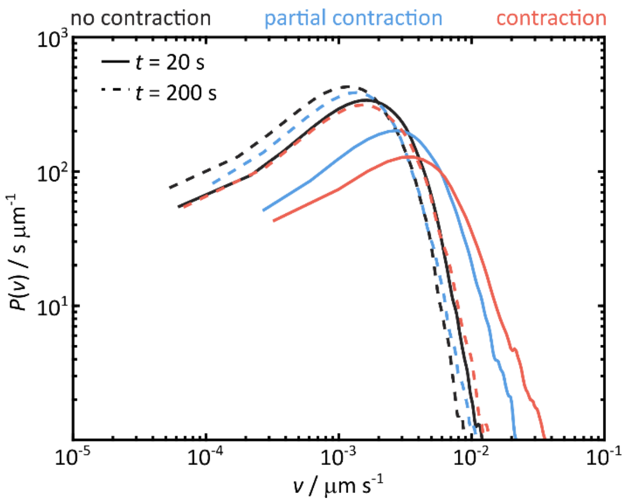

Figure S5. Mean velocity magnitude distribution depending on F-actin network contractility. Comparison of the F-actin network contraction velocity 20 s (solid lines) and 200 s (dashed lines) after myosin II addition. Images were analyzed via PIV, for all non-contracting, partially contracting, and fully contracting networks. For the analysis, *n* time series from individual preparations were used. No contraction ($n = 19$), partial contraction ($n = 7$), and full contraction ($n = 9$).

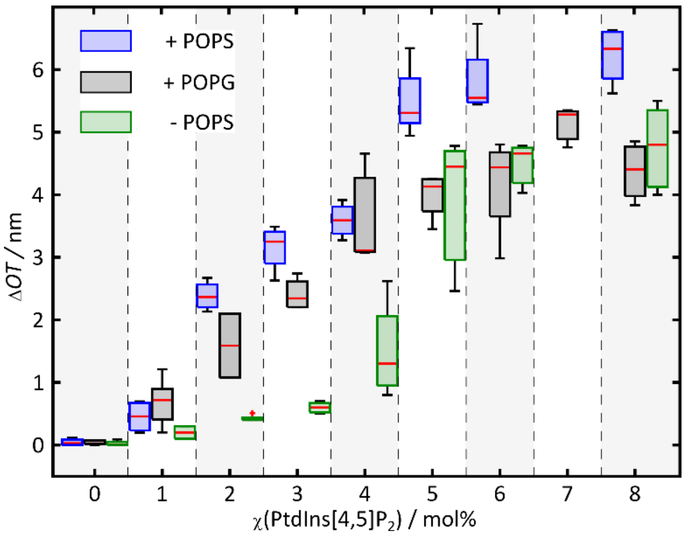

Figure S6. Comparison of the ezrin T567D binding as a function of the negatively charged lipids POPS and POPG. Change in optical thickness (Δ*OT*) caused by ezrin T567D binding to SLBs (POPC, green) doped with either 17 mol% POPS (blue) or 17 mol% POPG (black) as a function of the PtdIns[4,5]P_2 content. For the analysis *m* experiments were performed. 0 mol% ($m = 4$, $m = 3$, $m = 4$), 1 mol% ($m = 4$, $m = 6$, $m = 2$), 2 mol% ($m = 4$, $m = 2$, $m = 5$), 3 mol% ($m = 4$, $m = 4$, $m = 5$), 4 mol% ($m = 4$, $m = 3$, $m = 4$), 5 mol% ($m = 6$, $m = 4$, $m = 3$), 6 mol% ($m = 4$, $m = 4$, $m = 3$), 7 mol% ($m = 4$) and 8 mol% ($m = 4$, $m = 4$, $m = 7$). The Δ*OT* values for POPC and POPC/POPS membranes with 1-8 mol% PtdIns[4,5]P_2 were reproduced from Figure 1C. Boxes range from 25th to 75th percentiles of the sample, while whiskers represent the most extreme data points not considered as outliers (red crosses). Medians are shown as horizontals within the boxes.

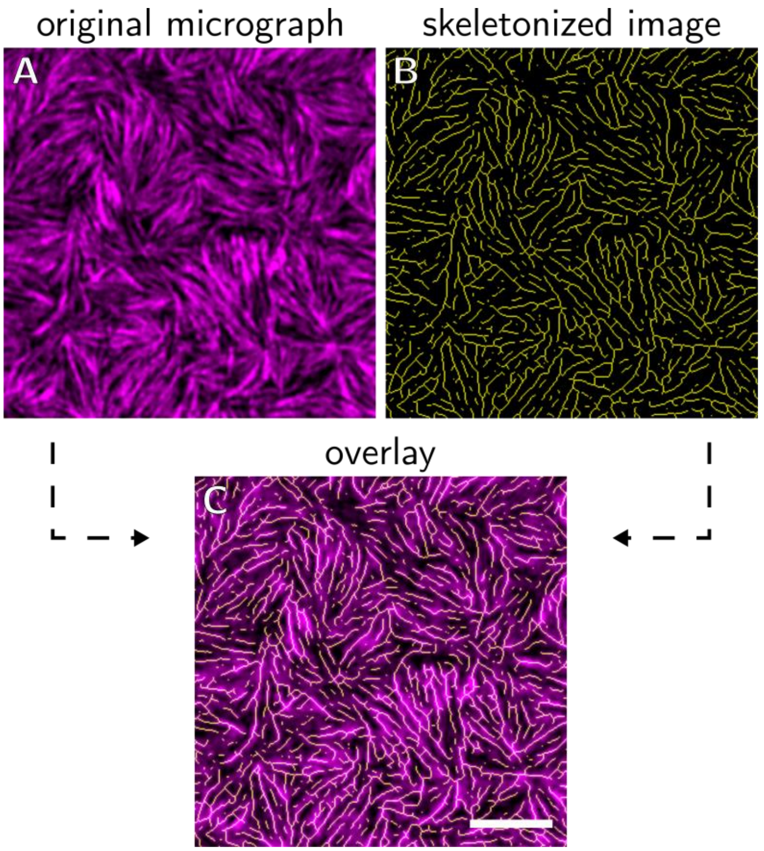

Figure S7. Skeleton-based actin intensity and relative bundling factor determination. (**A**) Exemplary fluorescence micrograph of a membrane-bound F-actin network (magenta) and (**B**) of the corresponding skeletonized image (yellow). (**C**) Overlay of the images shown in **A** and **B**. Actin fluorescence intensity was read out at the overlapping positions and averaged over the complete micrograph. The relative bundling factor was determined by normalizing the averaged actin intensity with the mean F-actin intensity at $\chi(\text{PtdIns}[4,5]\text{P}_2) = 1$ mol% without POPS.

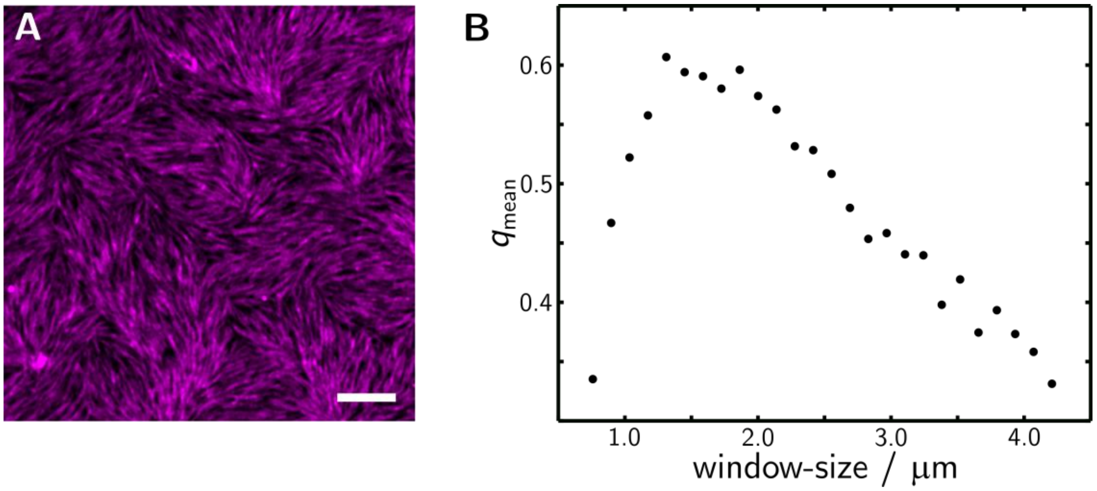

Figure S8. Determination of the optimal window size for the nematic order parameter. (**A**) Fluorescence micrograph of an exemplary F-actin network (magenta). (**B**) Mean nematic order parameter (q_{mean}) of the F-actin network shown in **A** as a function of the applied alignment vector field window size. For the exemplary image in **A** an optimal window size of 1.3 μm (q_{mean} ~0.6) was determined. Scale bar: 5 μm.

Table S1. PIV settings for the calculation of the F-actin velocity magnitude.

**Parameter**	
CLAHE window-size	50 px
PIV algorithm	FFT window deformation
Integration area:	
pass	64 px, step 32 px
pass	32 px, step 16 px
pass	16 px, step 8 px
Velocity limit	1 $\mu m\ s^{-1}$

Movies

Movie S1. Exemplary time series showing a non-contractile F-actin network (magenta) after the addition of myosin II (green, 0 s). Membrane composition: POPC/PtdIns[4,5]P_2/ATTO 390-DPPE (96.6:3:0.4).

Movie S2. Exemplary time series showing a contractile F-actin network (magenta) after the addition of myosin II (green, 0 s). Membrane composition: POPC/PtdIns[4,5]P_2/POPS/ATTO 390-DPPE (79.6:3:17:0.4).

Movie S3. Exemplary time series showing a partial contractile F-actin network (magenta) after the addition of myosin II (green, 0 s). Membrane composition: POPC/PtdIns[4,5]P_2/POPS/ATTO 390-DPPE (79.6:3:17:0.4).
